# Association of neutrophil to lymphocyte ratio with bone mineral density in post-menopausal women: a systematic review and meta-analysis

**DOI:** 10.1186/s12905-024-03006-1

**Published:** 2024-03-09

**Authors:** Maryam Salimi, Monireh Khanzadeh, Seyed Ali Nabipoorashrafi, Seyed Arsalan Seyedi, Shirin Yaghoobpoor, Jean-Michel Brismée, Brandon Lucke-Wold, Mehrnoosh Ebadi, Arshin Ghaedi, Varun Singh Kumar, Peyman Mirghaderi, Hamid Rabie, Shokoufeh Khanzadeh

**Affiliations:** 1 Bone and Joint Diseases Research Center, Department of Orthopedic Surgery, Shiraz University of Medical Sciences, Shiraz, Iran; 2 Geriatric & Gerontology Department, Medical School, Tehran University of medical and health sciences, Tehran, Iran; 3 Endocrinology and Metabolism Research Center (EMRC), School of Medicine, Vali-Asr Hospital, Tehran, Iran; 4 Student Research Committee, Faculty of Medicine, Shahid Beheshti University of Medical Sciences, Tehran, Iran; 5 Center for Rehabilitation Research, Department of Rehabilitation Sciences, Texas Tech University Health Sciences Center, Lubbock, TX USA; 6 Department of Neurosurgery, University of Florida, Gainesville, USA; 7 Faculty of Medicine, Arak University of Medical Sciences, Arak, Iran; 8 Student Research Committee, School of Medicine, Shiraz University of Medical Sciences, Shiraz, Iran; 9 Department of Orthopaedic Surgery, Ohio State University Wexner Medical Center, Columbus, OH USA; 10 Students’ Scientific Research Center (SSRC), Tehran University of Medical Sciences, Tehran, Iran; 11 Department of Orthopedic Surgery, Tehran University of Medical Sciences, Tehran, Iran; 12 Tabriz University of Medical Sciences, Tabriz, Iran

**Keywords:** Neutrophil to lymphocyte ratio, NLR, Post-menopausal osteoporosis, Meta-analysis

## Abstract

**Background:**

We conducted a systematic review and meta-analysis to compare the neutrophil lymphocyte ratio (NLR) levels between women with post-menopausal osteopenia or osteoporosis to those with normal bone mineral density (BMD).

**Methods:**

We used Web of Science, PubMed, and Scopus to conduct a systematic search for relevant publications published before June 19, 2022, only in English language. We reported standardized mean difference (SMD) with a 95% confidence interval (CI). Because a significant level of heterogeneity was found, we used the random-effects model to calculate pooled effects. We used the Newcastle–Ottawa scale for quality assessment.

**Results:**

Overall, eight articles were included in the analysis. Post-menopausal women with osteoporosis had elevated levels of NLR compared to those without osteoporosis (SMD = 1.03, 95% CI = 0.18 to 1.88, *p* = 0.017, I^2^ = 98%). In addition, there was no difference between post-menopausal women with osteopenia and those without osteopenia in neutrophil lymphocyte ratio (NLR) levels (SMD = 0.58, 95% CI=-0.08 to 1.25, *p* = 0.085, I^2^ = 96.8%). However, there was no difference between post-menopausal women with osteoporosis and those with osteopenia in NLR levels (SMD = 0.75, 95% CI=-0.01 to 1.51, *p* = 0.05, I^2^ = 97.5%, random-effect model).

**Conclusion:**

The results of this study point to NLR as a potential biomarker that may be easily introduced into clinical settings to help predict and prevent post-menopausal osteoporosis.

**Supplementary Information:**

The online version contains supplementary material available at 10.1186/s12905-024-03006-1.

## Background

Osteoporosis is a metabolic bone disease that affects about 10% of the world’s population [1]. It is far more common in postmenopausal women and to a lesser extent man over 70 years of age [2, 3]. Postmenopausal osteoporosis (PMO), the most common type of osteoporosis, which closely relates to estrogen deficiency, is marked by bone loss and micro-architectural destruction, resulting in bone fragility and higher risk of fracture [4].

Inflammation plays an important role within bone remodeling and osteoporosis development [5]. Inflammatory signals modulate bone production and degradation by activating osteoclasts with surrounding cytokines [6]. PMO is more common in inflammatory disorders such as ankylosing spondylitis, ulcerative colitis, systemic lupus erythematosus, rheumatoid arthritis, and Crohn’s disease, drawing attention to the link between PMO and chronic inflammation [7–9]. C-reactive protein (CRP), interleukin 6 (IL-6) and tumor necrosis factor-alfa (TNF-α) levels have been reported to be higher in PMO patients [10]. Berlunglundh et al. [11], on the other hand, reported that while CRP was not seen as a predictor of osteoporosis in older women, the highest CRP level was associated with PMO-related mortality.

The neutrophil is described as a cell that can perform functions other than those of a prototypical inflammatory cell, such as its ability to directly stimulate osteoclasts [12]. In numerous cancers and inflammatory disorders, the blood neutrophil lymphocyte ratio (NLR) Has been used as a non-invasive, cost-effective, and simple measure of inflammation [13, 14]. Up to now, the definite correlation between NLR and bone mineral density (BMD) has not been established.

Therefore, we conducted a systematic review and meta-analysis study to compare the NLR levels between women with post-menopausal osteopenia or osteoporosis to those with normal BMD. The findings of this study can serve to validate NLR as a marker of disease while also elucidating pathophysiology and advancing diagnostic modalities. To the best of our knowledge, this is the first systematic review and meta-analysis in this context.

## Materials and methods

### Study design and eligibility criteria

This study was conducted according to the Preferred Reporting Items for Systematic Reviews and Meta-analyses (PRISMA) 2020 reporting guideline [15]. We searched databases of PubMed, Web of Science, and Scopus up to June 19, 2022. In our literature search, we included a combination of keywords of NLR, neutrophil to lymphocyte ratio, Osteopenia, osteoporosis, post-menopause in the form of all field words or medical subject headings. The exact search strategy is detailed in Supplementary file A.

Additionally, we reviewed the reference lists of included and relevant studies to identify further eligible studies. Our inclusion criteria were based on the following PICO terms:

- Population: Women with post-menopausal osteopenia or osteoporosis.
- Intervention: NLR.
- Control: Post-menopausal women with normal BMD.
- Outcomes. The diagnostic performance of NLR.
- Study design: cohort, case-control, and cross-sectional studies.

Our exclusion criteria were as followed: (1) review articles, editorials/letters, case series, case reports, abstracts, and randomized controlled trials; (2) duplicate studies; (3) non peer-reviewed publications. There were no limitations on language or date of publication.

### Data extraction and quality assessment

The first author, year of publication, study design, study location, total sample size, number of cases and controls, mean and SD of NLR level, and any data for estimating the mean and SD (median and IQR or/and range) were all extracted. Two authors conducted the quality assessment of included studies, utilizing the Newcastle–Ottawa scale (NOS). This included three components: selection of the cohort, comparability of cohorts based on the design or analysis, how the exposure was ascertained, and how the outcomes of interest were assessed [16]. Disagreements between the authors were resolved via consensus. Those studies with six or more points were deemed to have good quality (reference).

### Certainty of evidence

The Grading of Recommendations Assessment, Development, and Evaluation (GRADE) method was used to assess the certainty of the evidence for the outcomes investigated in our study (osteoporosis and osteopenia) [17].

### Data synthesis and analysis

We performed the meta-analysis by using Stata 11.2 software (Stata Corp, College Station, TX). We used standardized mean difference (SMD) with a 95% confidence interval (CI) to compare the NLR level between cases and controls. The I^2^ and Cochran’s Q tests were adopted to determine the heterogeneity of the included studies. Significant heterogeneity between studies was conceived as I ^2^ >50% and *p*-value of the Q test < 0.05. Finally, because a significant level of heterogeneity was found, we applied the random-effects model to calculate pooled effects. In order to determine the publication bias, we used Egger test.

## Results

### Search results and included studies

**Fig. 1:**
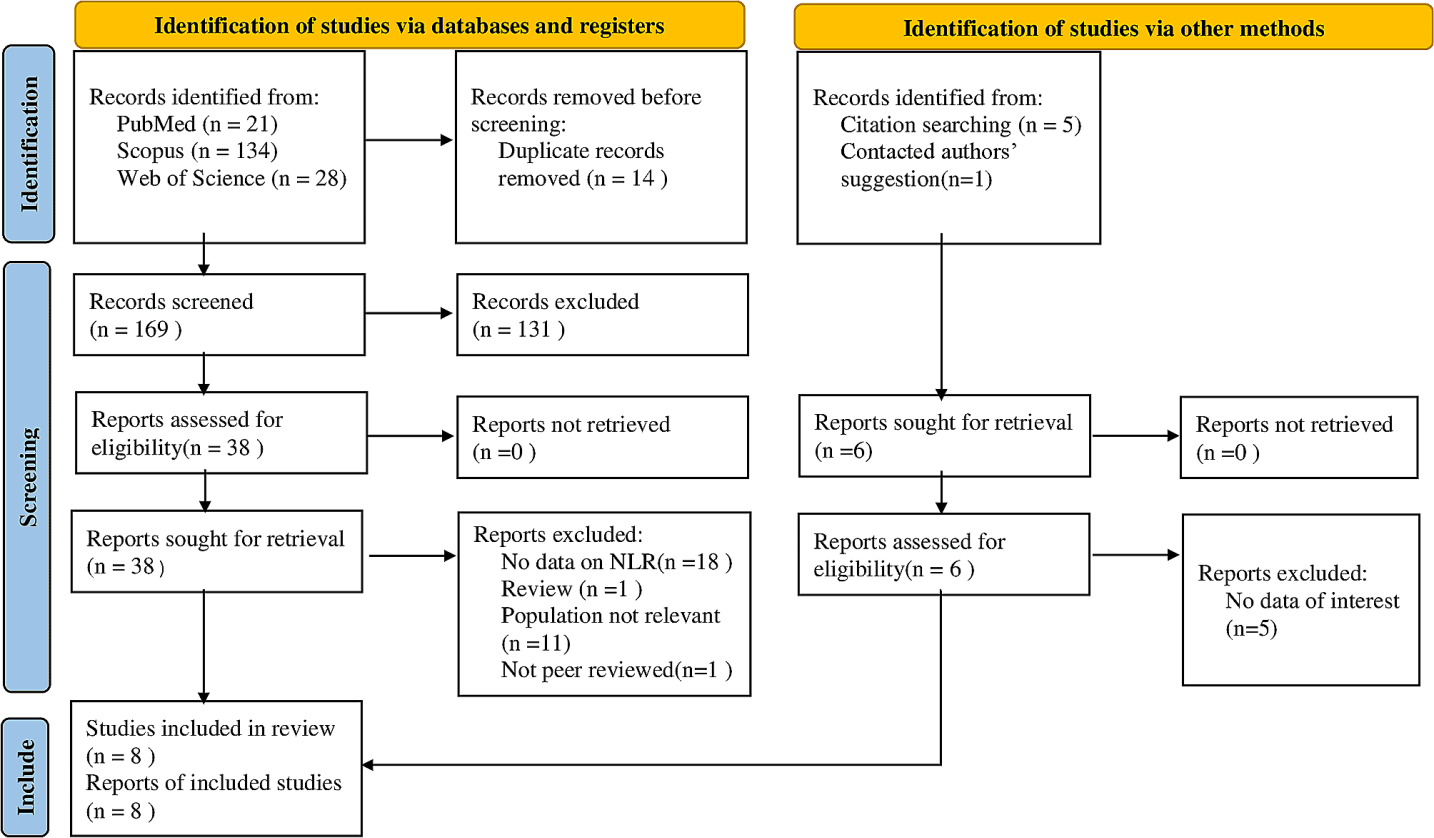
PRISMA 2020 Flow diagram for new systematic reviews which includes searches of databases, registers and other sources

The database search and manual search of the article citation list yielded a total of 324 results. Finally, eight papers were included in this systematic review and meta-analysis [18–25] after duplicates and non-relevant records were removed. Figure 1 shows the PRISMA flow diagram, indicating the process of inclusion and exclusion in details.

### Characteristics of the population and quality assessment

In total, eight articles were included in the analysis [18–25]. Six of them were written in English [18–20, 22–25] and one in Chinese [21]. There were four retrospective studies [18, 19, 21, 22] and four prospective studies [20, 23–25]. Four studies were conducted in China [20, 21, 23, 25], three in Turkey [19, 22, 24], and one in Oman [18]. Seven articles compared NLR Level of women with post-menopausal osteopenia to those with normal BMD [18–24], including 810 cases and 548 controls. In addition, seven articles reported NLR Level of women with PMO compared to those with normal BMD [18–22, 24, 25], including 871 cases and 628 controls. Also, six studies reported the differences in NLR level between women with PMO and those with post-menopausal osteopenia [18–22, 24], including 667 women with PMO and 669 women with post-menopausal osteopenia. Table 1 shows the overall characteristics of the included articles. The quality assessment revealed that all studies were of moderate to high quality based on the NOS scale (Table 1).

**Table 1:** General characteristics of included studies

First author	Year	Country	Design	Osteopenia		Osteoporosis		Normal BMD		NOS score
				N	NLR	N	NLR	N	NLR	
Yilmaz [24]	2014	Turkey	Prospective	152	3.17 ± 0.43	151	4.68 ± 0.72	135	2.10 ± 0.54	8
Liu [23]	2015	China	Prospective	141	3.00 ± 0.98			128	2.10 ± 0.77	8
Yu [25]	2015	China	Prospective			204	2.53 ± 0.65	208	2.09 ± 1.17	7
Huang [20]	2016	China	Prospective	60	2.55 ± 1.15	112	2.74 ± 1.06	51	2.12 ± 0.89	6
Eroglu [19]	2019	Turkey	Retrospective	112	2.28 ± 0.96	48	3.28 ± 1.81	92	2.58 ± 1.12	6
Kale [22]	2021	Turkey	Retrospective	103	1.67 ± 0.63	48	1.91 ± 0.74	26	1.47 ± 0.41	6
Salmani [18]	2021	Oman	Retrospective	164	1.17 ± 0.95	221	1.19 ± 1.05	65	1.22 ± 0.64	7
Huifang [21]	2022	China	Retrospective	78	2.06 ± 0.61	87	2.52 ± 0.82	51	1.81 ± 0.49	6

NLR: Neutrophil to lymphocyte ratio; NOS: Newcastle-Ottawa scale; BMD: Bone mineral density

### NLR Level in women with post-menopausal osteoporosis

**Fig. 2:**
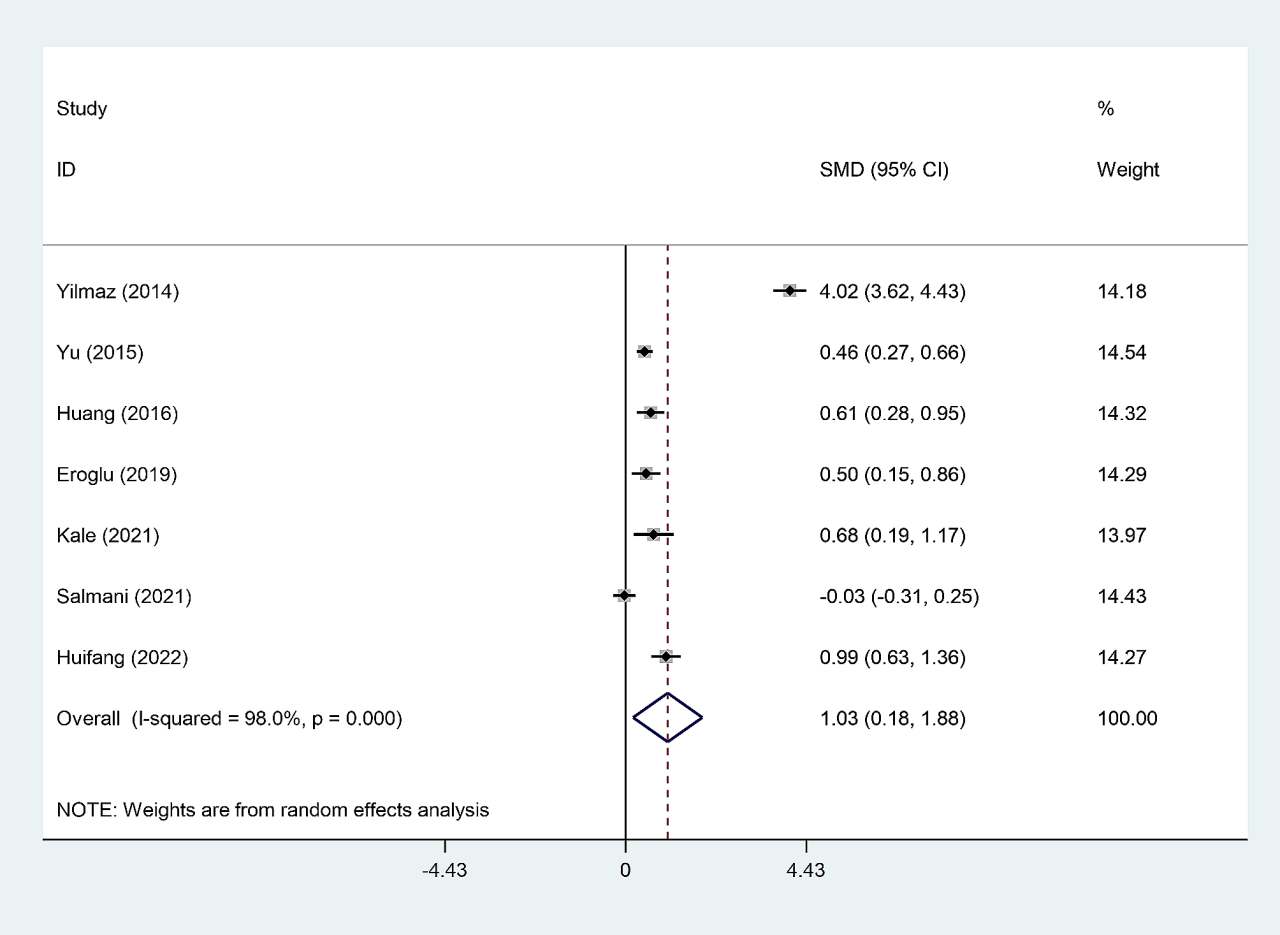
Meta-analysis of differences in NLR level between post-menopausal women with osteopenia and those without osteoporosis

A random-effect model revealed that post-menopausal women with osteoporosis had elevated levels of NLR compared to those without osteoporosis (SMD = 1.03, 95% CI = 0.18 to 1.88, p = 0.017) (Fig. 2). However, the certainty of evidence was very low in this analysis (Table 2).

**Table 2:** GRADE^1^ Evidence Profile for studies on the association of NLR with BMD in post-menopausal women

Certainty assessment							№ of patients		Certainty^7^	Importance
№ of studies	Study design	Risk of bias^2^	Inconsistency^3^	Indirectness^4^	Imprecision^5^	Publication bias^6^	Participants, n	Cases, n		
**Osteoporosis**										
7	observational studies	not serious	very serious	not serious	not serious	none	1499	871	⨁◯◯◯ Very low	CRITICAL
**Osteopenia**										
7	observational studies	not serious	very serious	not serious	not serious	none	1358	810	⨁◯◯◯ Very low	CRITICAL

^1^Grading of Recommendations Assessment, Development and Evaluation

^2^Risk of bias based on Newcastle-Ottawa Scale

^3^When I^2^ was < 30% inconsistency considered as Not serious limitation, > 50 considered as serious and more than 75% considered as very serious limitation

^5^Serious limitations when there was fewer than 4000 participants for each outcome and very serious limitations when there was fewer than 300 participants for each outcome

^6^Funnel plot revealed no asymmetry; neither test of publication bias approached *P* < 0.10

^7^Data from cohort studies begin with a grade of “LOW”. Downgraded for very serious inconsistency

**Fig. 3:**
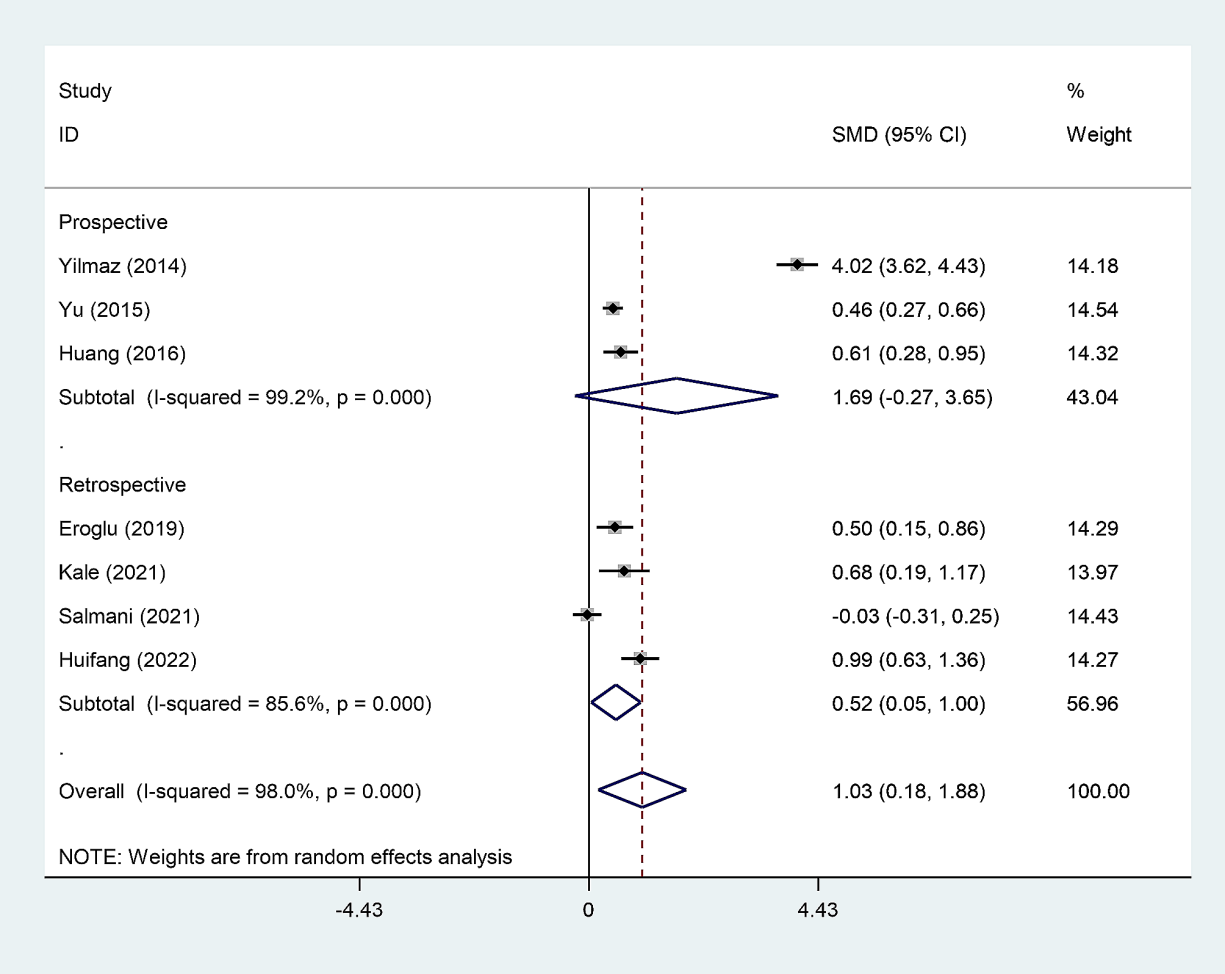
Subgroup analysis of differences in NLR level between post-menopausal women with osteopenia and those without osteoporosis, according to study design

In the subgroup analysis according to study design, we found that post-menopausal women with osteoporosis had elevated levels of NLR compared to those without osteoporosis in retrospective studies, but not in prospective studies (Fig. 3).

**Fig. 4:**
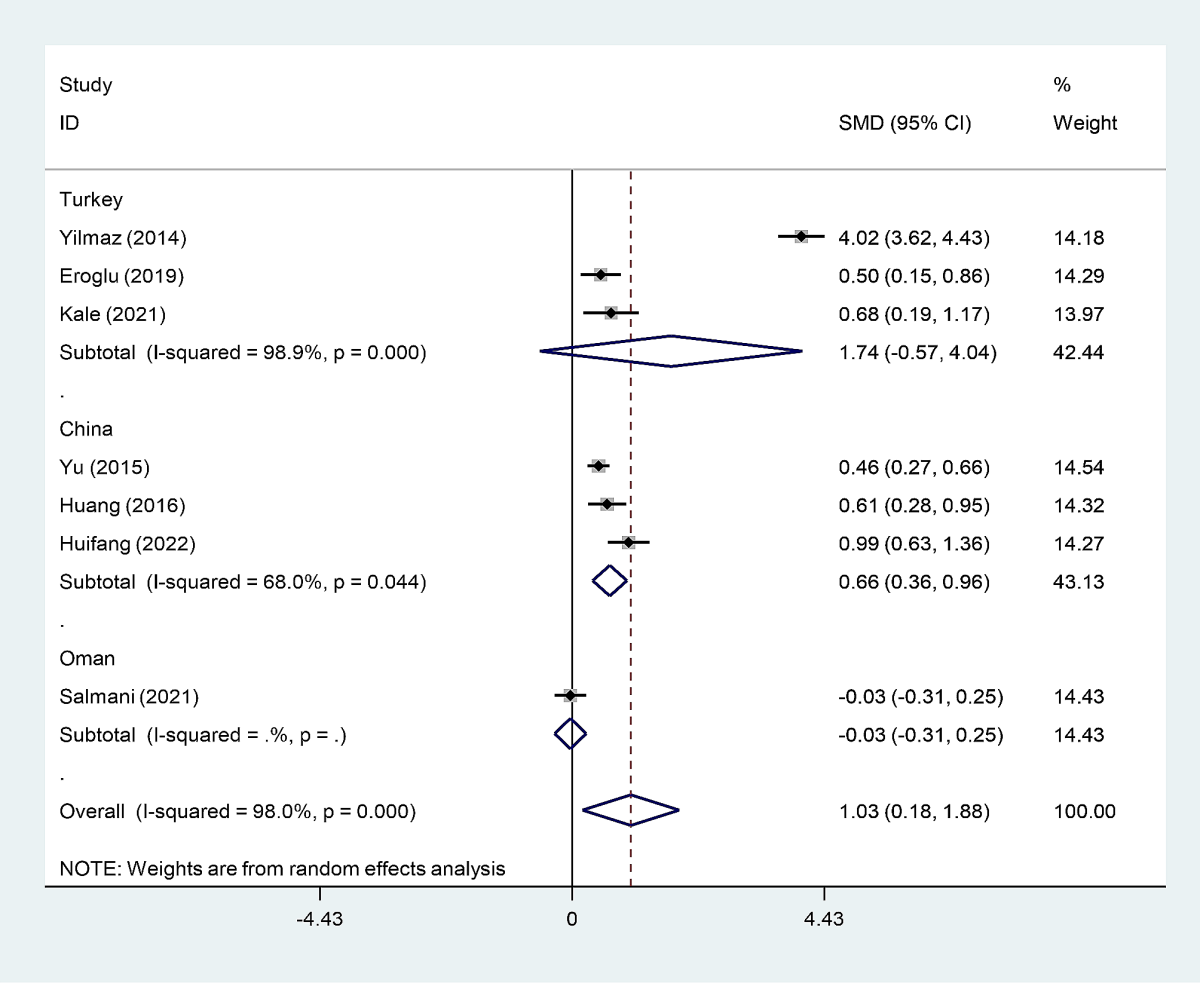
Subgroup analysis of differences in NLR level between post-menopausal women with osteopenia and those without osteoporosis, according to study location

In the subgroup analysis according to study location, we found that post-menopausal women with osteoporosis had elevated levels of NLR compared to those without osteoporosis in China, but not in Turkey or Oman (Fig. 4).

### NLR Level in women with post-menopausal osteopenia

**Fig. 5:**
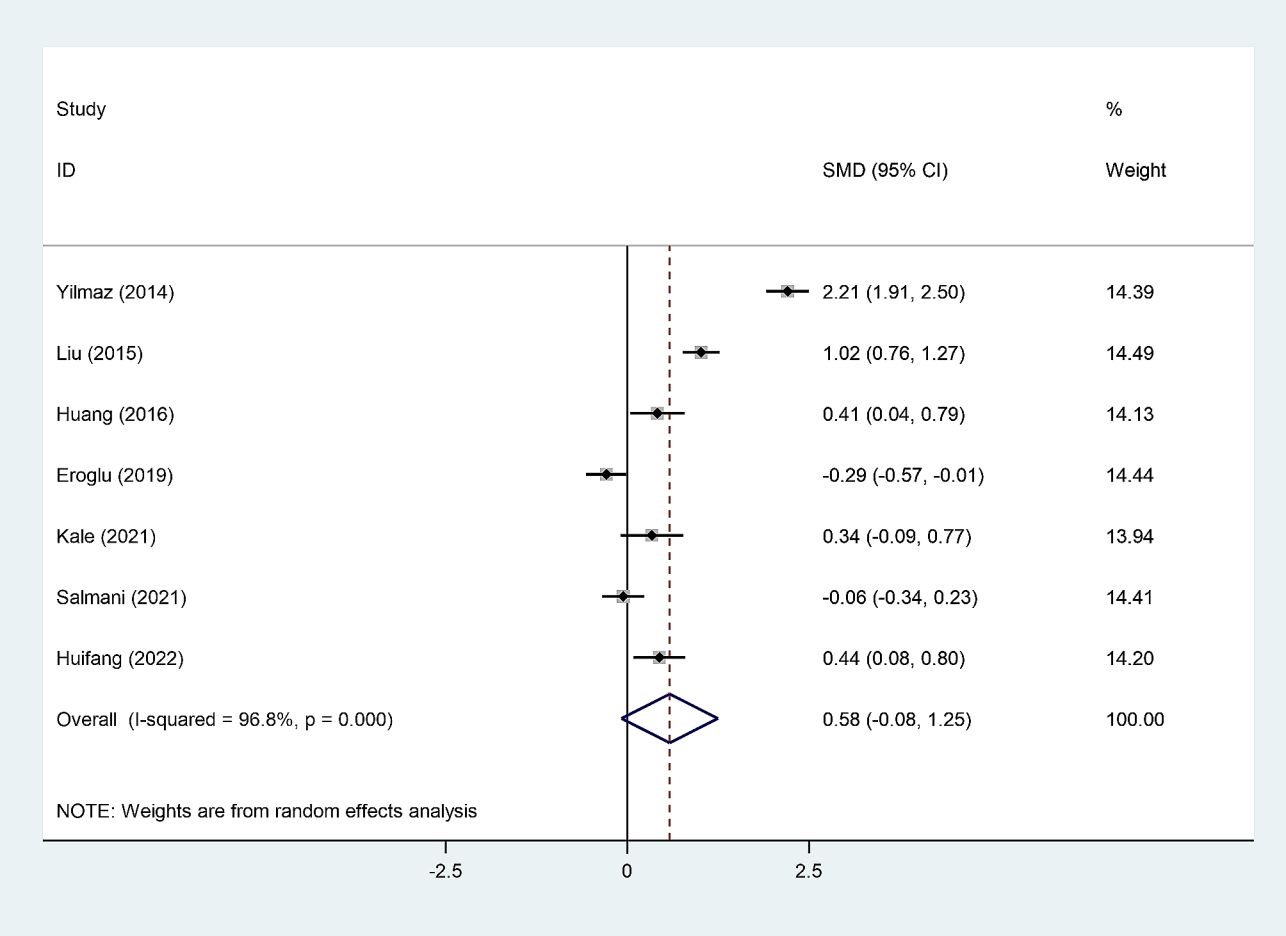
Meta-analysis of differences in NLR level between post-menopausal women with osteopenia and those without osteopenia

A random-effect model revealed that there was no difference between post-menopausal women with osteopenia and those without osteopenia in NLR levels (Fig. 5). The certainty of this summary estimate of effect was very low according to the GRADE approach (Table 2).

**Fig. 6:**
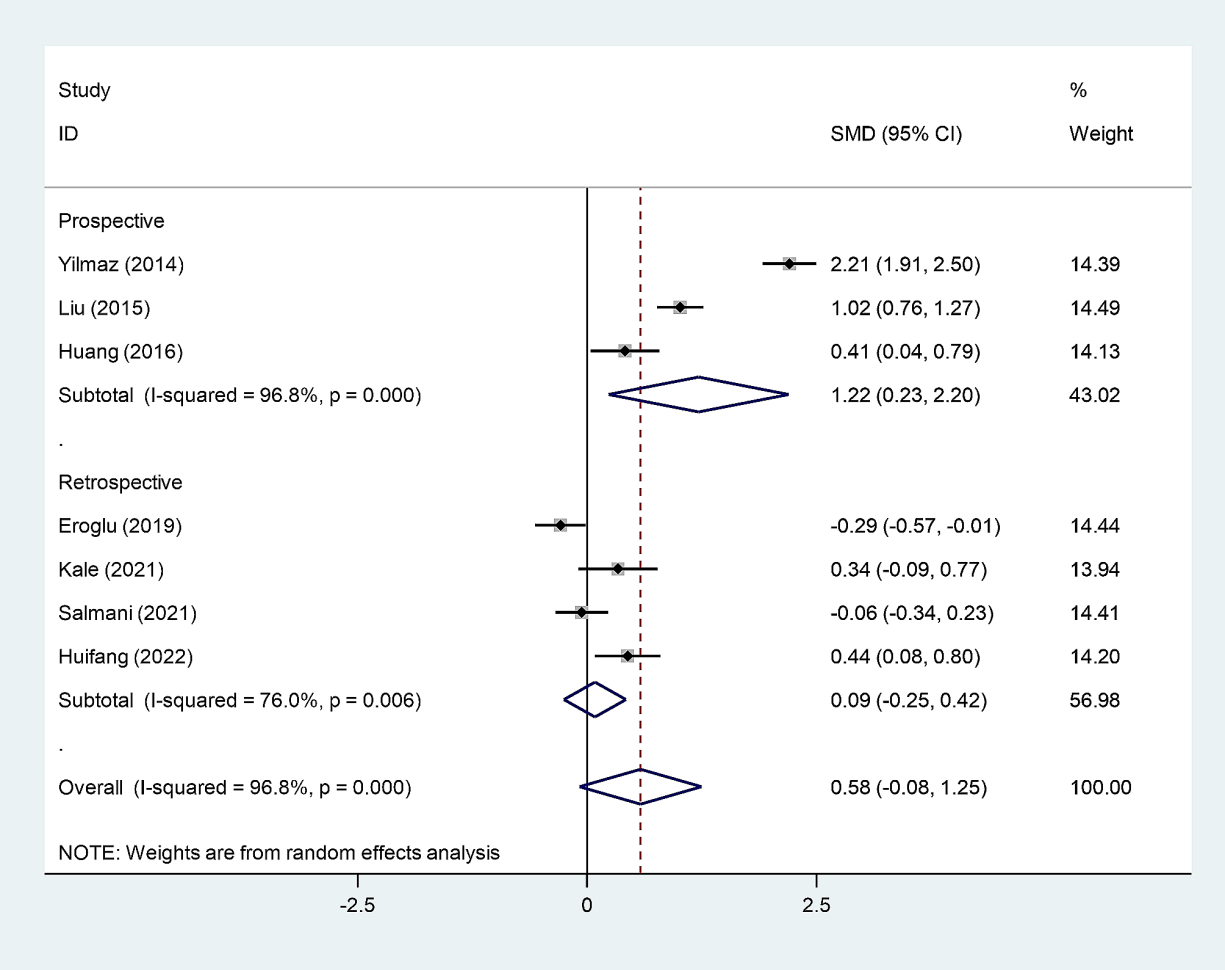
Subgroup analysis of differences in NLR level between post-menopausal women with osteopenia and those without osteopenia, according to study design

In the subgroup analysis according to study design, we found that post-menopausal women with osteopenia had elevated levels of NLR compared to those without osteopenia in prospective studies, but not in retrospective studies (Fig. 6).

**Fig. 7:**
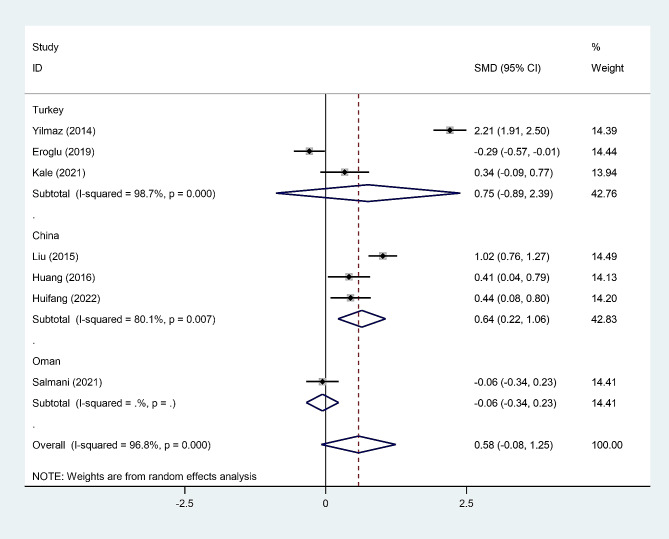
Subgroup analysis of differences in NLR level between post-menopausal women with osteopenia and those without osteopenia, according to study location

In the subgroup analysis according to study location, we found that post-menopausal women with osteopenia had elevated levels of NLR compared to those without osteopenia in China, but not in Turkey or Oman (Fig. 7).

### Differences in NLR Level between women with post-menopausal osteoporosis and those with osteopenia

**Fig. 8:**
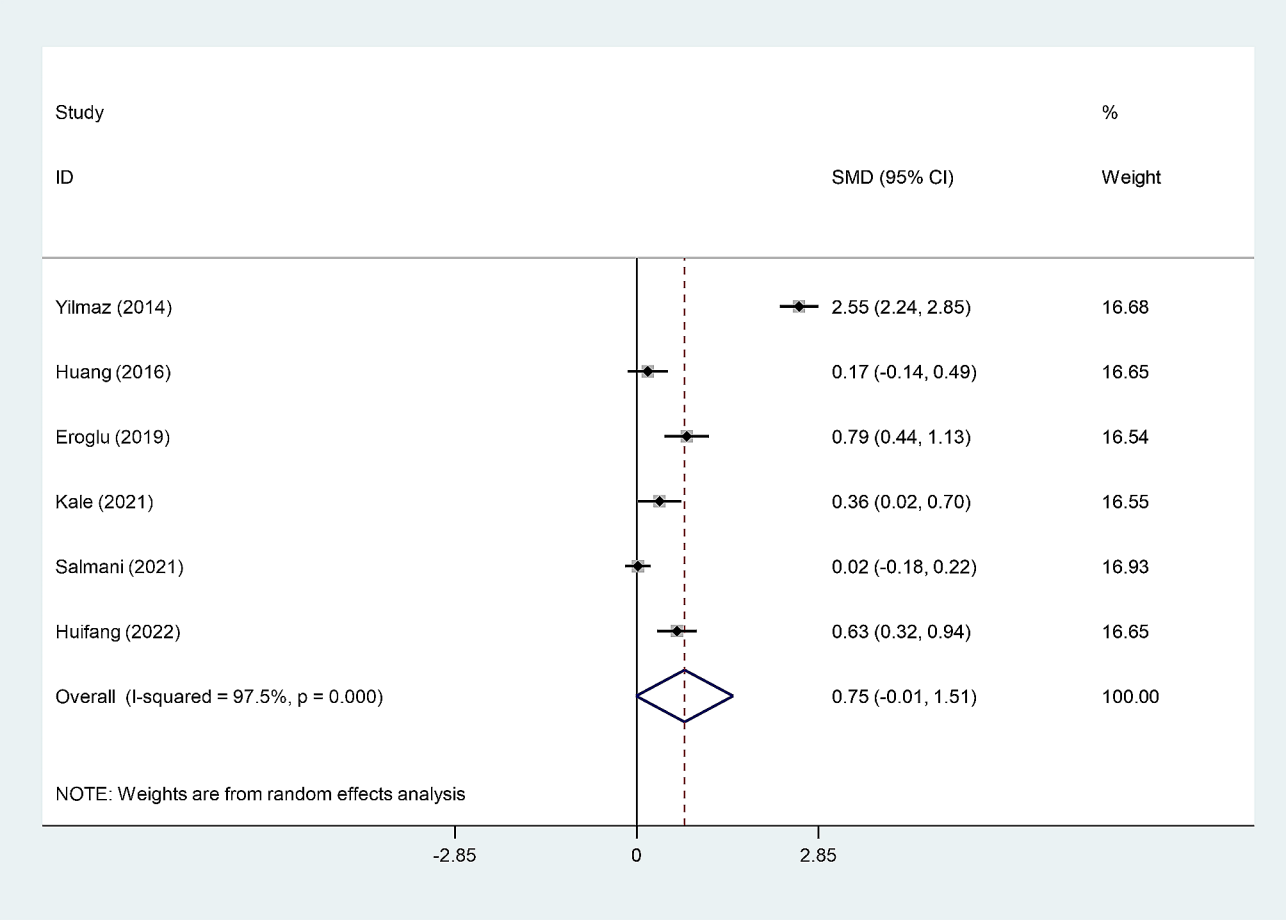
Meta-analysis of differences in NLR level between post-menopausal women with osteoporosis and those with osteopenia in NLR levels

As illustrated in Fig. 8, there were no differences between post-menopausal women with osteoporosis and those with osteopenia utilizing NLR levels.

### Publication bias

There was no significant publication bias among either studies on osteopenia (Egger’s test *p* = 0.70) or studies on osteoporosis (Egger’s test *p* = 0.36).

## Discussion

In the current systematic review and meta-analysis, we combined eight studies to investigate whether there was a significant difference in levels of NLR between PMO patients and post-menopausal women without osteoporosis. We found that post-menopausal women with osteoporosis had significantly elevated levels of NLR compared to those without osteoporosis. However, our meta-analysis did not detect a significant difference between post-menopausal women with osteopenia and those without osteopenia in NLR levels. It appears that once the process of osteopenia begins, the proinflammatory state becomes apparent. The NLR serves as a good initial marker with others having been associated in the literature such as IL-6 and TNF-alpha.

Postmenopausal osteoporosis is known as a systemic illness defined by reduced bone mass and degradation of bone microarchitecture, increasing the risk of fracture [26]. When estrogen levels drop after menopause, the balance between bone production and bone resorption shifts in favor of bone resorption [26]. One reason is estrogen’s direct impact on bone cells. Estrogen enhances bone production by increasing osteoblast maturation and osteogenic differentiation of mesenchymal stem cells (MSCs). Furthermore, estrogen reduces bone resorption by inhibiting osteoclast production and inducing osteoclast death. When estrogen levels within a woman’s body are low, these osteo-anabolic and anti-osteoclastic actions are suppressed, resulting in continued bone loss [27]. Because PMO is a complicated disorder involving the entire body, estrogen does not simply affect bone cells and thus cause PMO. Estrogen interacts with a variety of immune cells, resulting in a chronic low degree pro-inflammatory condition for estrogen-deficient individuals [28–30]. Since interactions between immune cells and bone cells occur at a variety of levels, it is plausible to assume that bone loss in menopause women partially stems from interactions between the immune cells and bone metabolism. To explain the mechanisms underlying higher levels of NLR in PMO patients, it is required to figure out the roles that neutrophils and lymphocytes play in this disease. It is a topic of ongoing investigation and pre-clinical models have indicated the oxidative stress and endoplasmic reticulum stress is often increased in ovariectomized mice as well. Whether this is seen in humans is yet to be determined.

In our study, the postmenopausal women displayed higher NLR. Changes in neutrophil levels during the menstrual cycle [31] and enhanced neutrophil infiltrations during inflammatory processes in ovariectomized (OVX) mice [32–35] provide evidence of direct estrogen effects on neutrophils. Estrogen has been shown to affect neutrophil chemotaxis, activity, apoptosis, and the generation of NO and ROS in vitro [36–38]. Overall, neutrophils may have a role in the development of PMO since estrogen affects their quantity, activity, and roles, and they release mediators that stimulate osteoclastic bone resorption, such as IFN-γ, IL-6, and receptor activator of nuclear factor kappa-Β ligand (RANKL). In a study by Moutsopoulos et al. on a periodontitis model, insufficient neutrophil recruitment to inflamed gingiva caused Th17 cells to release more IL-17, which is known to increase osteoclastic bone resorption [39]. This indirectly verifies the impact of neutrophils on osteoclasts. Activated neutrophils in rheumatoid arthritis have been demonstrated to produce RANKL, which induces osteoclastic bone resorption within the inflamed joint [40, 41]. It is worth noting that RANKL is also strongly expressed in the neutrophils of chronic obstructive pulmonary disease patients, who typically have osteoporosis and a decline in bone mineral density [42]. In bone biopsies of osteomyelitis patients with bone erosions, activated neutrophils were also discovered. Higher number of osteoclasts and elevated expression level of IL-8 have been demonstrated to stimulate osteoclast formation [43]. These were associated with infiltrated neutrophils. In an in vitro model of chronic gouty arthritis, neutrophils were found to directly adhere to osteoblasts causing osteoblast retraction without impacting osteoblastic matrix mineralization while increasing osteoclastic matrix resorption [44].

Active neutrophils appear to cause osteoclast production both directly and indirectly in inflammatory circumstances. On the other hand, a shortage of neutrophils has an impact on bone, as individuals who have severe chronic neutropenia have reduced bone mineral density, which is likely due to accelerated bone turnover and production of the pro-inflammatory cytokines including IL-1 and TNF [45]. As a result, senescent neutrophils are crucial for bone homeostasis, but highly active neutrophils may play role in the occurrence of bone loss. As an in vitro co-culture model of neutrophils, osteoblasts, and endothelial cells indicated that neutrophils promote the expression of osteogenic markers such as alkaline phosphatase, osteocalcin, collagen type 1, transforming growth factor-beta (TGF-β) and bone morphogenetic protein (BMP) in osteoblasts, neutrophils also affect osteoblasts. Furthermore, osteoblastic mineral deposition was enhanced, showing that neutrophils may have an osteogenic effect in bone [46]. Since mesenchymal stem cells (MSCs) co-cultured with activated neutrophils developed into osteoblasts, it shows that they are influenced by changing cytokine levels of IL-1 and TGF [47]. Neutrophils furthermore have an impact on MSCs. Further in vitro tests, indicated that neutrophils block MSCs from producing extracellular matrix factors [48]. G-CSF-induced neutrophil growth caused MSCs and osteoblasts to undergo apoptosis in vitro via neutrophil-produced ROS [49].

It is crucial to note that when there is a fracture hematoma, neutrophils are the first cells to enter the site of fracture and they phagocyte debris and cells and also secrete cytokine to draw in additional immune cells. After severe trauma, it has been shown that locally elevated neutrophil counts hinder bone healing, apparently under the direction of IL-6 [50]. Interestingly, neutrophil depletion also adversely affects the bone healing. So, balanced neutrophil functions seem to be a need for adequate fracture repair [51]. It is interesting to consider that Midkine-antibody therapy decreased IL-6 levels and neutrophil counts in OVX mice [52]. Midkine is an established pro-inflammatory cytokine that draws neutrophils in different inflammatory conditions and more importantly, it is an estrogen-regulated cytokine [53]. So, it might explain that when estrogen levels decrease in postmenopausal females, the regulatory effect of midkine on neutrophils would be impaired, while we observe a high number of neutrophils at fracture sites due to high midkine.

Also, there is compelling evidence that osteoblasts are a major source of activated complement proteins under inflammatory circumstances, which activate immune cells, and particularly neutrophils [54–56]. Certainly, the activation status of neutrophils may have a significant impact on osteogenic consequences. Finally, neutrophils produce and secrete inflammatory mediators that can impact osteoblasts, MSCs, and osteoclasts directly or indirectly. However, more research is required to understand the molecular mechanisms of cellular interactions in bone, especially in the absence of estrogen.

Lymphocytes are considered to play stimulatory or modulatory roles in osteoporosis. T cells are critical components of adaptive immunity [57–59]. During activation, they are exposed to various environmental stimuli (cytokines, antigens, etc.) and differentiate into diverse subpopulations. Furthermore, T cell-deficient animals displayed increased osteoclastogenesis and reduced bone mass, suggesting that T cells play a crucial role in maintaining bone homeostasis in vivo [60]. Other investigations have shown that inactivated T helper (Th) cells reduce osteoclast development [61]. This might be because Th cells do not release RANKL at steady-state circumstances [62]. T cell activation, on the other hand, leads to increased production of TNF-α and RANKL under inflammatory circumstances, encouraging osteoclastogenesis, different inflammatory processes, and eventual bone loss [63]. This is consistent with the findings of Peng et al. [64]; an elevation in TNF-α could exacerbate osteoporosis in the enrolled population. Stopping the inflammatory cascade at any point, on the other hand, significantly lowers bone loss [65]. These findings demonstrate that aberrant T lymphocyte numbers may result in altered bone metabolism [66, 67]. Environmental cytokines seem to alter the development of CD4 + cells into Th1 and Th2 cells [57, 59]. Furthermore, it has been observed that Th2 dominance is related with senile osteoporosis [66, 68], implying that Th2-type cytokines such as IL-10, IL-6, IL-5, and IL-4 levels rose while Th1-type cytokines such as IL-2,IFN-γ and TNF-α reduced in patients suffering from senile osteoporosis. Conversely, Peng et al. discovered that TNF-α was raised in the osteoporosis group, which is likely attributable to TNF-α release by other immune cells [64]. Meanwhile, decreased Th1-type cytokine release inhibits CD8 + T cell proliferation and activation, and subsequently lowering the CD8 + T lymphocytes numbers [64]. CD8 + T cells are an important component of the adaptive immune system, and they play a key role in immunological protection against intracellular microorganisms such as bacteria, viruses, and other diseases like cancers [69–71]. CD8 + T lymphocytes have a role in bone metabolism, and they suppress osteoclast development by secreting soluble proteins like osteoprotegerin (OPG) [72]. CD8 + T cells have also been found to protect the bone against metastases under bone tumor burdens in recent years [73].

On the other hand, B lymphocytes have represented active regulatory effects on the RANK/RANKL/OPG system, which is recognized to play a critical effector function in bone homeostasis, osteoclast production, and bone resorption control [74]. B cells generate active mediators for bone maintenance from early B-cell development in the bone marrow to the plasma cell stage, and they also have a number of regulatory cytokines and chemokines, as well as their receptors and downstream signaling molecules, in common with bone-forming and bone-resorbing cells [75]. Human B cells have been shown to secrete the anti-osteoclastogenic factor, OPG [76], despite the fact that osteoblasts have long been thought to be the principal source of OPG. A study with a mouse model, indicated that the main source of OPG in a mouse were B lineage cells in bone marrow under physiological conditions [60]. In line, B-cell knock out mice were discovered to be osteoporotic and deficient in bone marrow OPG; but both OPG deficiency and osteoporosis were reversed by reintroducing B cells to them [77]. When comparing women with osteoporosis to healthy controls, Breuil et al. discovered significantly lower quantity of CD19 + B lymphocytes and, more notably, the size of several subpopulations of memory B cells were in women with osteoporosis [78]. The mentioned evidence can explain higher NLR among PMO patients that is confirmed by our meta-analysis.

The neutrophil count represents the body’s inflammatory state, while the lymphocyte count is influenced by stress and food [79]. Blood cells that contribute to inflammatory reactions include lymphocytes, thrombocytes, and neutrophils. Previous research has shown that peripheral lymphopenia, neutrophilia, and thrombocytosis reflect the overall inflammatory state of the body system. Low lymphocyte counts suggest inflammation, while high neutrophil counts imply persistent inflammation [80–82]. Lymphocytes contribute in the regulation of inflammation, while neutrophils assist in its persistence [83]. We know that neutrophil numbers increase and neutrophilia develops during inflammatory processes. It should be noted that lymphocytopenia generally follows neutrophilia [84]. Because lymphocyte numbers decrease as neutrophil levels rise, it is explainable to utilize the NLR value to assess the diagnosis or course of inflammatory illnesses such as PMO. The preceding sentences also explain why NLR levels rise in PMO sufferers.

### Strength and limitations

In the present meta-analysis, we collected all information on the relationship between NLR and PMO. Although a meta-analysis often improves the strength of the available evidence, there are several limitations that must be taken into account when evaluating the findings of our research. First off, the results might be impacted by the limited number of included studies and participants. Second, none of the included studies stated the blood analyzer instrument’s machine type or its reference ranges, which might have affected our findings. Third, we could only include a small number of papers in our meta-analysis. Ultimately, there was still heterogeneity among the included studies even though this meta-analysis was carried out using a random effect model and included subgroup analysis. Our findings’ generalizability was constrained by the substantial heterogeneity of the total pooled data, which had an overall I^2^ value of 96.8% and 98%.

We hypothesized that research design and location may be factors in heterogeneity, and subgroup analysis was performed to further investigate this. However, since heterogeneity did not reduce following subgroup analysis, such stratifications did not seem to explain it. As a result, we hypothesize that other variables, such as differences in osteoporosis diagnosis and study populations, may be driving the heterogeneity. Baseline NLR levels, for example, seem to differ by race [85, 86]. Such variations may indicate underlying differences in the degree to which NLR reacts to pathologic insults across different populations, potentially introducing further heterogeneity. Other confounding variables, such as smoking, age and drug use, might have influenced our findings. Furthermore, the research protocol had not been pre-registered for this review. This is a source of concern since it puts possible bias into the review.

Regardless of these limitations, our results have significant clinical implications. Blood NLR might be a handy and promising biomarker to anticipate osteopenia and osteoporosis in postmenopausal women. As far as we know, this is the first meta-analysis that thoroughly summarizes evidence concerning the connection between NLR and BMD in such patients. Other significant strengths of our meta-analysis should be mentioned as well. First, in addition to the manual reference search of the references of the first chosen publications, reviews, meta-analyses, or comments, we devised a systematic and repeatable search approach for each database. Furthermore, suitable subgroup analyses were done across studies, yielding almost consistent results.

## Conclusion

In conclusion, the findings of this systematic review and meta-analysis support the significant higher levels of NLR among PMO women in comparison with post-menopausal women without osteoporosis. Therefore, NLR could be used in clinics as a potential predictor to aid physicians in the detection of PMO among post-menopausal women. Further research is needed to conduct a meta-analysis with higher number of included studies to attain more exact results.

### Electronic supplementary material

Below is the link to the electronic supplementary material.

Supplementary Material 1

## Data Availability

All data generated or analysed during this study are included in this published article.

## Abbreviations

NLR: Neutrophil to lymphocyte ratio
SMD: Standardized mean difference
SD: Standard deviation
95% CI: 95% confidence interval
N: Number
NOS: The Newcastle-Ottawa Quality Assessment Scale
R: Retrospective
P: Prospective
PRISMA: Preferred Reporting Items for Systematic Reviews and Meta-Analyses
BMD: Bone mineral density
PMO: Postmenopausal osteoporosis
CRP: C-reactive protein
IL-6: Interleukin 6
TNF-α: Tumor necrosis factor-alfa
MSCs: Mesenchymal stem cells
OVX: Ovariectomized
RANKL: Receptor activator of nuclear factor kappa-Β ligand
TGF-β: Transforming growth factor-beta
MSCs: Mesenchymal stem cells
Th: T helper
OPG: Osteoprotegerin
