# Supplementary material for: Association of neutrophil to lymphocyte ratio with bone mineral density in post-menopausal women: a systematic review and meta-analysis
Source: BMC Womens Health. 2024 Mar 9;24:169. doi: 10.1186/s12905-024-03006-1 (PMC10924380; doi:10.1186/s12905-024-03006-1)
Supplement: Supplementary file 1 — Supplementary Material 1 [file 12905_2024_3006_MOESM1_ESM.docx]

**Title**

**Relationship between neutrophil to lymphocyte ratio and bone mineral density among post-menopausal women: a systematic review and meta-analysis**

Search strategy

Scopus

N=96

( ALL ( "Neutroohil to lymphocyte ratio" OR nlr ) AND ALL ( postmenopaus* OR post-menopaus* ) AND ALL ( osteoporosis OR osteopeni* ) )

ProQuest

N=193

("Neutrophil to lymphocyte ratio" OR NLR) AND (Osteopeni* OR osteoporosis and Postmenopaus* OR Post-menopaus*)

Web of science

N=13

("Neutrophil to lymphocyte ratio" OR NLR) AND (Osteopeni* OR osteoporosis and Postmenopaus* OR Post-menopaus*)

PubMed

N=15

("Neutrophil to lymphocyte ratio"[All Fields] OR "NLR"[All Fields]) AND ((("osteopeni*"[All Fields] OR "osteoporosis"[All Fields]) AND ("postmenopaus*"[All Fields] OR "post menopaus*"[All Fields])) OR "osteoporosis, postmenopausal"[MeSH Terms])
